# *N*-(4-Meth­oxy-2-nitro­phen­yl)-*N*-(methyl­sulfon­yl)acetamide

**DOI:** 10.1107/S1600536809011799

**Published:** 2009-04-02

**Authors:** Muhammad Zia-ur-Rehman, Amir Sepehrianazar, Muhammad Ali, Waseeq Ahmad Siddiqui, Nagihan Çaylak

**Affiliations:** a Applied Chemistry Research Centre, PCSIR Laboratories Complex, Lahore 54600, Pakistan; b Department of Chemical Engineering, Islamic Azad University, Ahar Branch, Ahar-54516, Iran; c Department of Chemistry, University of Sargodha, Sargodha, Pakistan; d Department of Physics, Sakarya University, Sakarya, Turkey

## Abstract

In the title compound, C_10_H_12_N_2_O_6_S, the nitro group is twisted slightly out of the plane of the aromatic ring, forming a dihedral angle of 20.79 (1)°.  In the crystal, the mol­ecules arrange themselves as a chain along the *a* axis through inter­molecular C—H⋯O inter­actions.

## Related literature

For the synthesis of sulfur-containing heterocyclic compounds, see: Siddiqui *et al.* (2007 ▶); Wen *et al.* (2006 ▶); Zhang *et al.* (2006 ▶). For related structures, see: Zhang *et al.* (2006 ▶); Wen *et al.* (2005 ▶); Zia-ur-Rehman *et al*. (2008 ▶).
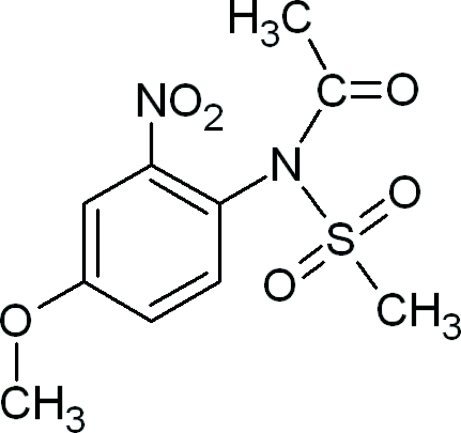


## Experimental

### 

#### Crystal data

C_10_H_12_N_2_O_6_S
*M* *_r_* = 288.29
Monoclinic, 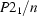

*a* = 7.1512 (2) Å
*b* = 15.4303 (5) Å
*c* = 11.3217 (3) Å
β = 91.769 (2)°
*V* = 1248.70 (6) Å^3^
*Z* = 4
Mo *K*α radiation
μ = 0.29 mm^−1^
*T* = 296 K
0.21 × 0.11 × 0.08 mm

#### Data collection

Bruker APEXII CCD area-detector diffractometer
Absorption correction: multi-scan (*SADABS*; Bruker, 2007 ▶) *T*_min_ = 0.958, *T*_max_ = 0.974
14122 measured reflections
3102 independent reflections
2101 reflections with *I* > 2σ(*I*)
*R*_int_ = 0.042

#### Refinement

*R*[*F* ^2^ > 2σ(*F* ^2^)] = 0.044
*wR*(*F* ^2^) = 0.128
*S* = 1.05
3102 reflections
172 parameters
H-atom parameters constrained
Δρ_max_ = 0.30 e Å^−3^
Δρ_min_ = −0.32 e Å^−3^

### 

Data collection: *APEX2* (Bruker, 2007 ▶); cell refinement: *SMART* (Bruker, 2007 ▶); data reduction: *SAINT* (Bruker, 2007 ▶); program(s) used to solve structure: *SHELXS97* (Sheldrick, 2008 ▶); program(s) used to refine structure: *SHELXL97* (Sheldrick, 2008 ▶); molecular graphics: *PLATON* (Spek, 2009 ▶) and *Mercury* (Macrae *et al.*, 2006 ▶); software used to prepare material for publication: *SHELXTL* (Sheldrick, 2008 ▶) and local programs.

## Supplementary Material

Crystal structure: contains datablocks I, global. DOI: 10.1107/S1600536809011799/bt2918sup1.cif

Structure factors: contains datablocks I. DOI: 10.1107/S1600536809011799/bt2918Isup2.hkl

Additional supplementary materials:  crystallographic information; 3D view; checkCIF report

## Figures and Tables

**Table 1:** Hydrogen-bond geometry (Å, °)

*D*—H⋯*A*	*D*—H	H⋯*A*	*D*⋯*A*	*D*—H⋯*A*
C10—H10*B*⋯O3^i^	0.96	2.50	3.453 (3)	169

Symmetry code: (i) 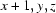.

**Fig. 1.**
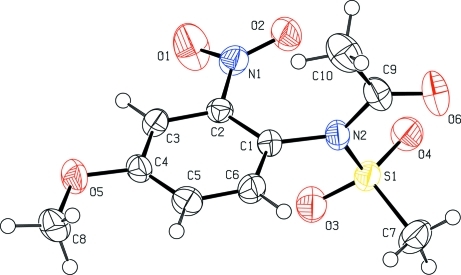
The asymmetric unit of the title compound. Displacement ellipsoids are drawn at the 50% probability level.

**Fig. 2.**
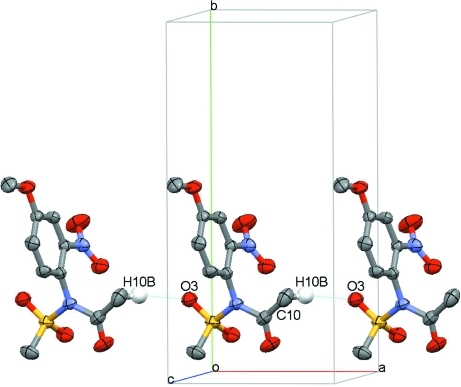
Perspective view of the crystal packing showing inter molecular C—H···O interactions (dashed lines) along a. H atoms not involved in hydrogen bonding have been omitted for clarity.

## supplementary crystallographic information

### Comment

*N*-(Substituted phenyl)acetamides are considered as important intermediates in organic synthesis. A large number of heterocyclic compounds such as 2,5-piperazinedione (Wen *et al.*, 2006), (quinolin-8-yloxy) acetamide (Zhang  *et al.*, 2006) and 2,2-(1,3,4-thiadiazolyl-2,5-dithio)diacetamide (Wen *et al.*, 2005) are being efficiently synthesized starting from such acetamides. In the present paper, the structure of *N*-(4-Methoxy-2-nitrophenyl)-*N*-(methylsulfonyl)acetamide has been determined as part of a research program involving the synthesis and biological evaluation of sulfur containing heterocyclic compounds (Siddiqui *et al.*, 2007).

In the molecule  (Fig. 1), the bond lengths and bond angles are similar to those in the related molecules (Wen *et al.*, 2006; Zhang *et al.*, 2006) and are within in normal ranges. The nitro group is slightly twisted out of the plane of the aromatic ring. Each molecule is linked to its neighbour by  inter molecular C—H···O interactions forming a chain along the *a* axis (Table 1 and Fig. 2).

### Experimental

A mixture of *N*-(4-methoxy-2-nitrophenyl)methane sulfonamide (2.462507 g; 10.0 mmoles) and acetic anhydride (10.0 ml) was heated to reflux for half an hour and then poued over crushed ice. Resultant solids were then washed with cold water and dried under reduced pressure. Yellow crystals were obtained by slow evaporation of an ethanolic solution over a period of two days.

### Refinement

H atoms bound to C were placed in geometric positions (C—H distance = 0.93 to 0.96 Å) using a riding model with *U*_iso_(H) = 1.2 *U*_eq_(C) or *U*_iso_(H) = 1.5 *U*_eq_(C_methyl_).

### Crystal data

**Table:**

C_10_H_12_N_2_O_6_S	*F*(000) = 600
*M**_r_* = 288.29	*D*_x_ = 1.533 Mg m^−^^3^
Monoclinic, *P*2_1_/*n*	Mo *K*α radiation, λ = 0.71073 Å
Hall symbol: -P 2yn	Cell parameters from 3173 reflections
*a* = 7.1512 (2) Å	θ = 2.6–26.8°
*b* = 15.4303 (5) Å	µ = 0.28 mm^−^^1^
*c* = 11.3217 (3) Å	*T* = 296 K
β = 91.769 (2)°	Needles, yellow
*V* = 1248.70 (6) Å^3^	0.21 × 0.11 × 0.08 mm
*Z* = 4	

### Data collection

**Table:**

Bruker APEXII CCD area-detector diffractometer	3102 independent reflections
Radiation source: fine-focus sealed tube	2101 reflections with *I* > 2σ(*I*)
graphite	*R*_int_ = 0.042
φ and ω scans	θ_max_ = 28.3°, θ_min_ = 2.6°
Absorption correction: multi-scan (*SADABS*; Bruker, 2007)	*h* = −8→9
*T*_min_ = 0.958, *T*_max_ = 0.974	*k* = −20→20
14122 measured reflections	*l* = −15→15

### Refinement

**Table:**

Refinement on *F*^2^	Primary atom site location: structure-invariant direct methods
Least-squares matrix: full	Secondary atom site location: difference Fourier map
*R*[*F*^2^ > 2σ(*F*^2^)] = 0.044	Hydrogen site location: inferred from neighbouring sites
*wR*(*F*^2^) = 0.128	H-atom parameters constrained
*S* = 1.05	*w* = 1/[σ^2^(*F*_o_^2^) + (0.0614*P*)^2^ + 0.2596*P*] where *P* = (*F*_o_^2^ + 2*F*_c_^2^)/3
3102 reflections	(Δ/σ)_max_ < 0.001
172 parameters	Δρ_max_ = 0.30 e Å^−^^3^
0 restraints	Δρ_min_ = −0.31 e Å^−^^3^

### Special details

**Table:**

Geometry. All e.s.d.'s (except the e.s.d. in the dihedral angle between two l.s. planes) are estimated using the full covariance matrix. The cell e.s.d.'s are taken into account individually in the estimation of e.s.d.'s in distances, angles and torsion angles; correlations between e.s.d.'s in cell parameters are only used when they are defined by crystal symmetry. An approximate (isotropic) treatment of cell e.s.d.'s is used for estimating e.s.d.'s involving l.s. planes.
Refinement. Refinement of *F*^2^ against ALL reflections. The weighted *R*-factor *wR* and goodness of fit *S* are based on *F*^2^, conventional *R*-factors *R* are based on *F*, with *F* set to zero for negative *F*^2^. The threshold expression of *F*^2^ > σ(*F*^2^) is used only for calculating *R*-factors(gt) *etc*. and is not relevant to the choice of reflections for refinement. *R*-factors based on *F*^2^ are statistically about twice as large as those based on *F*, and *R*- factors based on ALL data will be even larger.

### Fractional atomic coordinates and isotropic or equivalent isotropic displacement parameters (Å^2^)

**Table:**

	*x*	*y*	*z*	*U*_iso_*/*U*_eq_	
S1	0.23442 (8)	0.16533 (4)	0.86323 (4)	0.03679 (18)	
O1	0.3919 (3)	0.43383 (14)	0.73036 (15)	0.0788 (7)	
O2	0.5342 (3)	0.31646 (11)	0.76949 (14)	0.0535 (5)	
O3	0.0897 (2)	0.22746 (11)	0.84351 (15)	0.0493 (4)	
O4	0.3188 (2)	0.12704 (11)	0.76361 (13)	0.0489 (4)	
O5	0.1853 (2)	0.54616 (10)	1.10272 (14)	0.0473 (4)	
O6	0.6008 (3)	0.10620 (12)	0.94545 (17)	0.0634 (5)	
N1	0.4337 (3)	0.37563 (12)	0.79726 (15)	0.0390 (4)	
N2	0.3979 (2)	0.21872 (11)	0.94551 (14)	0.0353 (4)	
C1	0.3511 (3)	0.30400 (13)	0.98604 (17)	0.0311 (4)	
C2	0.3598 (3)	0.37840 (13)	0.91680 (16)	0.0296 (4)	
C3	0.3029 (3)	0.45779 (13)	0.95680 (17)	0.0334 (5)	
H3	0.3089	0.5064	0.9084	0.040*	
C4	0.2359 (3)	0.46491 (14)	1.07076 (18)	0.0341 (5)	
C5	0.2273 (3)	0.39268 (15)	1.14165 (18)	0.0413 (6)	
H5	0.1845	0.3973	1.2181	0.050*	
C6	0.2828 (3)	0.31336 (15)	1.09848 (18)	0.0393 (5)	
H6	0.2740	0.2647	1.1464	0.047*	
C7	0.1573 (4)	0.08423 (16)	0.9575 (2)	0.0538 (7)	
H7A	0.2552	0.0425	0.9707	0.081*	
H7B	0.0497	0.0561	0.9222	0.081*	
H7C	0.1245	0.1095	1.0316	0.081*	
C8	0.1068 (4)	0.55680 (17)	1.2166 (2)	0.0525 (7)	
H8A	0.0775	0.6168	1.2288	0.079*	
H8B	0.1955	0.5379	1.2766	0.079*	
H8C	−0.0053	0.5228	1.2209	0.079*	
C9	0.5736 (3)	0.18093 (16)	0.97108 (19)	0.0431 (6)	
C10	0.7164 (4)	0.23869 (19)	1.0292 (2)	0.0590 (7)	
H10A	0.6632	0.2951	1.0409	0.089*	
H10B	0.8226	0.2436	0.9798	0.089*	
H10C	0.7552	0.2146	1.1042	0.089*	

### Atomic displacement parameters (Å^2^)

**Table:**

	*U*^11^	*U*^22^	*U*^33^	*U*^12^	*U*^13^	*U*^23^
S1	0.0472 (4)	0.0292 (3)	0.0344 (3)	−0.0019 (2)	0.0066 (2)	−0.0012 (2)
O1	0.1238 (19)	0.0701 (14)	0.0443 (10)	0.0384 (13)	0.0293 (11)	0.0251 (10)
O2	0.0705 (12)	0.0439 (10)	0.0476 (9)	0.0051 (9)	0.0257 (8)	−0.0059 (8)
O3	0.0464 (10)	0.0414 (10)	0.0595 (10)	0.0024 (8)	−0.0069 (8)	−0.0045 (8)
O4	0.0640 (11)	0.0455 (10)	0.0378 (8)	−0.0032 (8)	0.0122 (7)	−0.0081 (7)
O5	0.0607 (11)	0.0338 (9)	0.0480 (9)	0.0058 (8)	0.0120 (8)	−0.0095 (7)
O6	0.0757 (14)	0.0435 (11)	0.0705 (12)	0.0253 (10)	−0.0037 (10)	−0.0059 (9)
N1	0.0486 (12)	0.0364 (11)	0.0324 (9)	−0.0029 (9)	0.0081 (8)	0.0001 (8)
N2	0.0418 (11)	0.0276 (10)	0.0367 (9)	0.0042 (8)	0.0042 (7)	−0.0011 (7)
C1	0.0328 (11)	0.0271 (11)	0.0335 (10)	0.0007 (9)	0.0034 (8)	−0.0016 (8)
C2	0.0292 (11)	0.0312 (11)	0.0285 (9)	−0.0022 (9)	0.0037 (7)	−0.0016 (8)
C3	0.0378 (12)	0.0283 (11)	0.0341 (10)	−0.0029 (9)	0.0017 (8)	0.0015 (8)
C4	0.0320 (12)	0.0313 (12)	0.0391 (11)	0.0008 (9)	0.0029 (8)	−0.0084 (9)
C5	0.0494 (14)	0.0428 (14)	0.0324 (10)	−0.0010 (11)	0.0126 (9)	−0.0037 (10)
C6	0.0499 (14)	0.0333 (12)	0.0353 (10)	0.0013 (10)	0.0103 (9)	0.0060 (9)
C7	0.0731 (18)	0.0406 (14)	0.0486 (13)	−0.0138 (13)	0.0156 (12)	0.0016 (11)
C8	0.0554 (16)	0.0489 (15)	0.0540 (14)	0.0012 (12)	0.0161 (12)	−0.0194 (12)
C9	0.0476 (14)	0.0426 (14)	0.0394 (11)	0.0143 (11)	0.0041 (10)	0.0010 (10)
C10	0.0478 (16)	0.0689 (19)	0.0597 (15)	0.0149 (14)	−0.0081 (12)	−0.0093 (14)

### Geometric parameters (Å, °)

**Table:**

S1—O3	1.4234 (17)	C3—H3	0.9300
S1—O4	1.4239 (15)	C4—C5	1.376 (3)
S1—N2	1.6868 (19)	C5—C6	1.381 (3)
S1—C7	1.745 (2)	C5—H5	0.9300
O1—N1	1.207 (2)	C6—H6	0.9300
O2—N1	1.209 (2)	C7—H7A	0.9600
O5—C4	1.357 (2)	C7—H7B	0.9600
O5—C8	1.432 (3)	C7—H7C	0.9600
O6—C9	1.206 (3)	C8—H8A	0.9600
N1—C2	1.469 (2)	C8—H8B	0.9600
N2—C9	1.407 (3)	C8—H8C	0.9600
N2—C1	1.437 (3)	C9—C10	1.493 (3)
C1—C6	1.385 (3)	C10—H10A	0.9600
C1—C2	1.393 (3)	C10—H10B	0.9600
C2—C3	1.372 (3)	C10—H10C	0.9600
C3—C4	1.394 (3)		
			
O3—S1—O4	118.63 (10)	C4—C5—H5	120.2
O3—S1—N2	104.23 (9)	C6—C5—H5	120.2
O4—S1—N2	109.67 (10)	C5—C6—C1	122.1 (2)
O3—S1—C7	109.68 (13)	C5—C6—H6	119.0
O4—S1—C7	109.68 (11)	C1—C6—H6	119.0
N2—S1—C7	103.83 (11)	S1—C7—H7A	109.5
C4—O5—C8	117.47 (18)	S1—C7—H7B	109.5
O2—N1—O1	122.44 (18)	H7A—C7—H7B	109.5
O2—N1—C2	119.68 (18)	S1—C7—H7C	109.5
O1—N1—C2	117.88 (18)	H7A—C7—H7C	109.5
C9—N2—C1	121.94 (18)	H7B—C7—H7C	109.5
C9—N2—S1	120.69 (15)	O5—C8—H8A	109.5
C1—N2—S1	117.36 (14)	O5—C8—H8B	109.5
C6—C1—C2	117.03 (19)	H8A—C8—H8B	109.5
C6—C1—N2	118.78 (18)	O5—C8—H8C	109.5
C2—C1—N2	124.09 (17)	H8A—C8—H8C	109.5
C3—C2—C1	122.09 (17)	H8B—C8—H8C	109.5
C3—C2—N1	116.68 (18)	O6—C9—N2	119.8 (2)
C1—C2—N1	121.22 (18)	O6—C9—C10	124.3 (2)
C2—C3—C4	119.31 (19)	N2—C9—C10	116.0 (2)
C2—C3—H3	120.3	C9—C10—H10A	109.5
C4—C3—H3	120.3	C9—C10—H10B	109.5
O5—C4—C5	125.15 (19)	H10A—C10—H10B	109.5
O5—C4—C3	114.93 (19)	C9—C10—H10C	109.5
C5—C4—C3	119.91 (19)	H10A—C10—H10C	109.5
C4—C5—C6	119.54 (19)	H10B—C10—H10C	109.5
			
O3—S1—N2—C9	−172.42 (16)	O1—N1—C2—C1	160.0 (2)
O4—S1—N2—C9	−44.42 (19)	C1—C2—C3—C4	0.7 (3)
C7—S1—N2—C9	72.73 (18)	N1—C2—C3—C4	−178.38 (18)
O3—S1—N2—C1	6.58 (17)	C8—O5—C4—C5	−3.8 (3)
O4—S1—N2—C1	134.58 (15)	C8—O5—C4—C3	177.00 (19)
C7—S1—N2—C1	−108.27 (16)	C2—C3—C4—O5	179.21 (19)
C9—N2—C1—C6	−85.2 (3)	C2—C3—C4—C5	−0.1 (3)
S1—N2—C1—C6	95.8 (2)	O5—C4—C5—C6	179.9 (2)
C9—N2—C1—C2	98.7 (2)	C3—C4—C5—C6	−0.9 (3)
S1—N2—C1—C2	−80.3 (2)	C4—C5—C6—C1	1.4 (4)
C6—C1—C2—C3	−0.3 (3)	C2—C1—C6—C5	−0.7 (3)
N2—C1—C2—C3	175.92 (19)	N2—C1—C6—C5	−177.2 (2)
C6—C1—C2—N1	178.73 (19)	C1—N2—C9—O6	172.7 (2)
N2—C1—C2—N1	−5.0 (3)	S1—N2—C9—O6	−8.3 (3)
O2—N1—C2—C3	158.5 (2)	C1—N2—C9—C10	−7.6 (3)
O1—N1—C2—C3	−21.0 (3)	S1—N2—C9—C10	171.38 (17)
O2—N1—C2—C1	−20.6 (3)		

### Hydrogen-bond geometry (Å, °)

**Table:**

*D*—H···*A*	*D*—H	H···*A*	*D*···*A*	*D*—H···*A*
C10—H10B···O3^i^	0.96	2.50	3.453 (3)	169

Symmetry codes: (i) *x*+1, *y*, *z*.
